# Operationalizing ecosystem service bundles for strategic sustainability planning: A participatory approach

**DOI:** 10.1007/s13280-020-01378-w

**Published:** 2020-09-18

**Authors:** Katja Malmborg, Elin Enfors-Kautsky, Cibele Queiroz, Albert Norström, Lisen Schultz

**Affiliations:** 1 Stockholm Resilience Centre, Stockholm University, Kräftriket 2B, 10691 Stockholm, Sweden; 2 Prosperous Planet, Sveavägen 131, 11346 Stockholm, Sweden

**Keywords:** Co-production of knowledge, Decision-support, Ecosystem service bundles, Multifunctional landscapes, Participatory methods, Social–ecological systems

## Abstract

The ecosystem service concept is recognized as a useful tool to support sustainability in decision-making. In this study, we collaborated with actors in the Helge å catchment, southern Sweden, in an iterative participatory ecosystem service assessment. Through workshops and interviews, we jointly decided which ecosystem services to assess and indicators to use in order to achieve a sense of ownership and a higher legitimacy of the assessment. Subsequently, we explored the landscape-level interactions between the 15 assessed services, and found that the area can be described using three distinct ecosystem service bundles. The iterative, participatory process strengthened our analysis and created a shared understanding and overview of the multifunctional landscape around Helge å among participants. Importantly, this allowed for the generated knowledge to impact local strategic sustainability planning. With this study, we illustrate how similar processes can support local decision-making for a more sustainable future.

**Electronic supplementary material:**

The online version of this article (10.1007/s13280-020-01378-w) contains supplementary material, which is available to authorized users.

## Introduction

Over the past decade, the ecosystem service concept has rapidly made its way into policy spheres. In 2012, the Intergovernmental Science-Policy Platform on Biodiversity and Ecosystem Services (IPBES) was launched, a UN wide effort to “strengthen the science-policy interface for biodiversity and ecosystem services for the conservation and sustainable use of biodiversity, long-term human well-being and sustainable development” (Díaz et al. 2015). At the EU level, many current environmental policies refer to ecosystem services, albeit with different interpretations of the concept (Bouwma et al. 2018). In Sweden, the national environmental quality objectives state that “by 2018, the importance of biodiversity and the value of ecosystem services are to be generally known and integrated into economic positions, political considerations and other decisions in society where it is relevant and reasonable to do so” (Ds 2012:23 2012). Locally, in Sweden and elsewhere, it has been incorporated into for example strategic and comprehensive plans for urban planning (Hansen et al. 2015; Schubert et al. 2018). In a Swedish context, human–nature interactions have been considered in strategic plans also prior to the introduction of the ecosystem service concept, but these considerations have been inconsistent over time (Wilkinson et al. 2013). Through the ecosystem service concept, its terminology and existing analytical frameworks, the importance of human–nature interactions can be addressed more systematically, making this broad policy uptake promising. It provides a window of opportunity for working towards sustainability in a concerted way (Wood et al. 2018).

Currently, many different interpretations of the ecosystem service concept exist, and a wide range of frameworks and approaches for operationalization have been developed. These range from those that are purely biophysical, quantitative and expert-led (Burkhard et al. 2012), to participatory mappings done with stakeholders (Malmborg et al. 2018). Of particular value for practical sustainability work, such as strategic planning, are social–ecological approaches that acknowledge that ecosystem services are produced and experienced through human–nature interactions (Daw et al. 2011; Reyers et al. 2013; Dunford et al. 2018; Vihervaara et al. 2019). However, these interactions can be weak or strong, depending on the service, and may occur at different scales. This implies that the methods used need to capture both social and ecological aspects of ecosystem service generation (van Oudenhoven et al. 2018). Otherwise, there is a risk that policies citing ecosystem services miss potential trade-offs and synergies either between types of services or beneficiaries (Daw et al. 2015; Cord et al. 2017), and as a consequence undermine rather than support sustainability.

One way to conceptualize social–ecological interactions in ecosystem service analyses is through a focus on bundles of ecosystem services (Foley 2005; Bennett et al. 2009). Ecosystem service bundles are sets of ecosystem services that co-occur across space or time (Raudsepp-Hearne et al. 2010). An ecosystem service bundles approach provides an analytical lens for characterizing landscapes according to their varying degrees of multifunctionality and for revealing trade-offs, synergies or mismatches between ecosystem services (Saidi and Spray 2018). During the past decade several studies have been published presenting different methods for identifying and analyzing ecosystem service bundles (Cord et al. 2017; Spake et al. 2017). These include spatially explicit analyses of current bundles of ecosystem service supply across landscapes (Raudsepp-Hearne et al. 2010; Queiroz et al. 2015) and countries (Turner et al. 2014), ecosystem service use (Hamann et al. 2015) and dynamics over time (Renard et al. 2015). These studies identify clear patterns of interactions between ecosystem services, for example re-occurring trade-offs between provisioning and regulating services (Raudsepp-Hearne et al. 2010; Turner et al. 2014). The benefit of these approaches is that they can help decision-makers avoid making interventions that enhance single ecosystem services while having unintended negative consequences for the generation of other important services (Queiroz et al. 2015). However, these studies also suggest that the intensity of interactions between services can vary depending on management practices (Raudsepp-Hearne et al. 2010). By identifying types of ecosystem service bundles, and common trade-offs and synergies, decision-makers can focus on potential desirable shifts between types, and develop strategic plans that support long-term sustainable development (Queiroz et al. 2015). An ecosystem service bundles approach can also provide powerful visualizations that give an overview and highlight areas in landscape management that need further attention or investigation, which can be used strategically in negotiations about priorities (McKenzie et al. 2014). Finally, these studies use publicly available data and a comparatively simple statistical clustering method, making them easy to replicate (Hamann et al. 2015). This further increases the usefulness of this approach for decision-making, since low-cost methods to increase understanding for the current state of the landscape can support later decisions to assign funding for more sophisticated analyses or primary data collection.

It has been emphasized recently, however, that in order to bridge the gap between research and decision-making, producing usable knowledge requires legitimate processes of co-production (Ruckelshaus et al. 2015; Clark et al. 2016). The people who, through their management, contribute to the generation of ecosystem services and those who benefit from them need to be involved in assessment and planning (Brunet et al. 2018; Dick et al. 2018). Further, situated knowledge, held by people in a particular landscape, is important for understanding how ecosystem services should be articulated and framed (Carmen et al. 2018). Finally, for the generated knowledge to actually be used and have an impact, it needs to fit with existing political and cultural contexts, which is easier to achieve if the knowledge is co-produced with the intended users themselves (Ruckelshaus et al. 2015; Norström et al. 2020). In a Swedish governance context, such knowledge co-production already exists to varying degrees, for example within and between the seven biosphere reserves (Olsson et al. 2007; Schultz et al. 2007; Heinrup and Schultz 2017). Municipalities in Sweden also have a high degree of autonomy, meaning that creating the structures for such processes is within their mandate (Johannessen and Hahn 2013). However, in these early stages of using the ecosystem service concept in decision-making, there is a need for support in translating theoretical knowledge from research into practical applications in municipal and regional plans and procedures (Schubert et al. 2018). Practitioners need to gain an understanding of the concept, so-called conceptual knowledge, before more instrumental uses can be applied meaningfully (Ruckelshaus et al. 2015). For this, a researcher-facilitated co-production process becomes particularly appropriate (McKenzie et al. 2014).

This paper draws on a case study from the Helge å catchment in southern Sweden. The Helge å catchment is a particularly interesting case, since many Swedish landscape types are represented there, from coniferous production forests in the north, to intense agriculture in the south. It also provides fertile ground for knowledge co-production, thanks to the history of adaptive co-management centered around the Kristianstad Vattenrike Biosphere Reserve (Hahn et al. 2006). In order to advance and test the usefulness of analyzing ecosystem service bundles for practitioners, we conducted a participatory ecosystem service assessment in the study area between 2015 and 2018. The paper describes how we went about to jointly define, describe and analyze the current landscape in the catchment from an ecosystem service bundles perspective together with a group of local and regional actors. Our guiding questions for the participatory ecosystem service assessment were the following: (1) How can the ecosystem service bundles analysis be advanced through an iterative participatory approach? (2) How has participating in such a process been useful for the participants?

## Background

### Description of study area—the Helge Å catchment

**Fig. 1:**
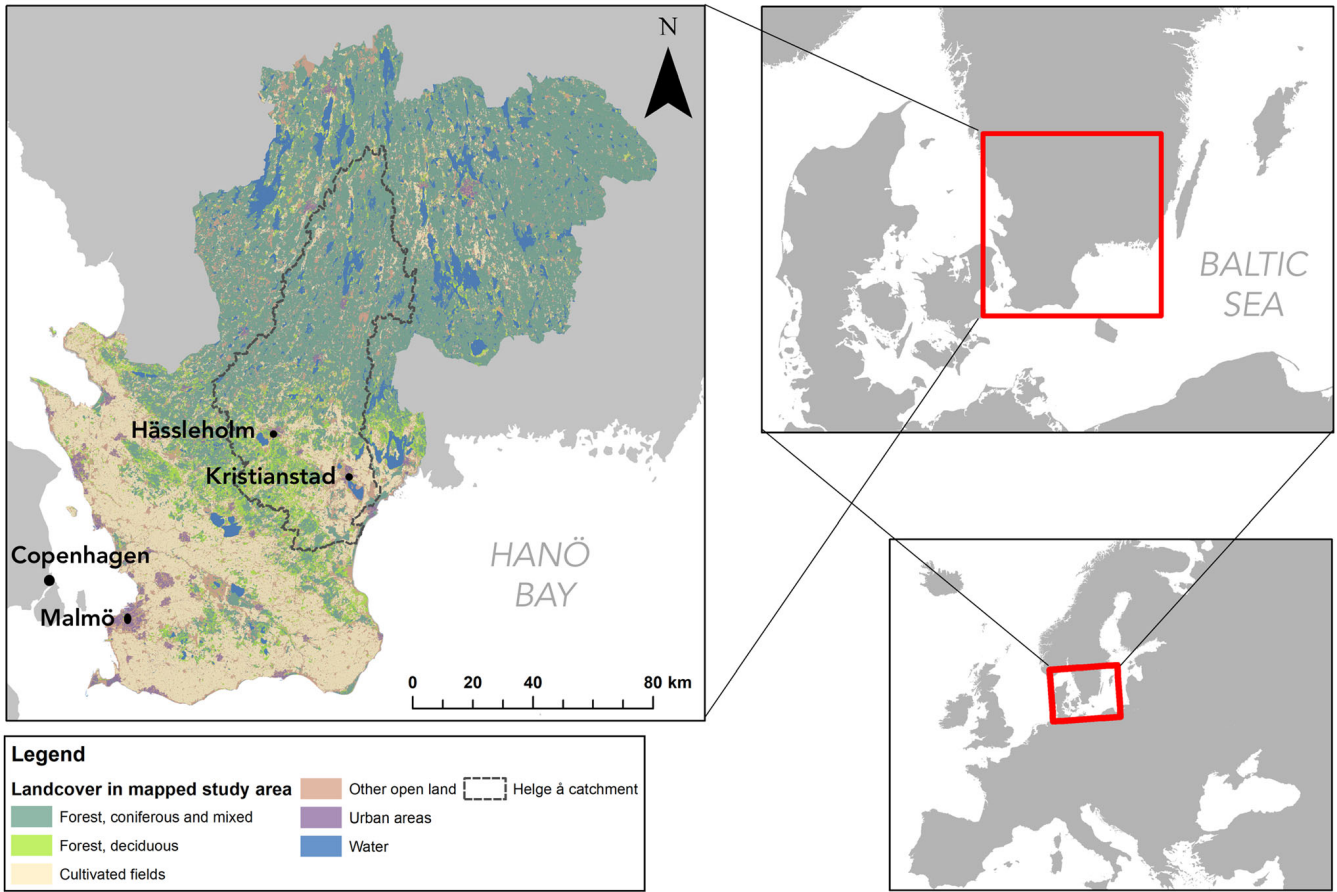
Map of case study area. The map to the right shows the general land covers in the mapped study area, based on the Swedish Land Survey economic map. The outline of the Helge å catchment is shown in the dashed gray line. To ensure the stability of the statistical analysis, the ecosystem service bundles analysis included all municipalities in the two counties that overlap with the Helge å catchment, Kronoberg and Skåne, as well as the municipality of Värnamo in Jönköping county (which overlaps with the northernmost tip of the catchment)

The Helge å catchment covers 4700 km^2^ in southern Sweden and spans 13 municipalities within Skåne, Kronoberg and Jönköping counties (Fig. 1). The upstream area is sparsely populated (14 inhabitants/km^2^) and mainly consists of coniferous production forests. The downstream area is more densely populated (62 inhabitants/km^2^) and consists of a mixed agricultural landscape (SCB 2018). In the south lie two mid-sized towns: Hässleholm and Kristianstad. Kristianstad municipality is also home to the Kristianstad Vattenrike Biosphere Reserve, an area known for its grazed flooded meadows with high biodiversity value. Here, adaptive co-management approaches were developed in the early 2000s, when the municipality, land owners and private citizens created new ways of working together to meet environmental challenges (Olsson et al. 2007).

Over the past 50 years, the Helge å catchment has changed substantially. The population has grown with 16 percent (SCB 2018) and the area has become dominated by larger scale and more homogenous farming and forestry enterprises. Brownification, a process of increasing levels of dissolved organic carbon in the river water due to a combination of changes in rain water acidity, forestry practices and climate change (Kritzberg et al. 2020), has emerged as an important issue, encompassing both upstream–downstream and exploitation–recreation conflicts (Tuvendal and Elmqvist 2011).

### Decision-support context of the research

When research is intended to be used for decision-making, it is important that its development is in response to a decision-support need (Marre and Billé 2019). Otherwise, there is a risk that it will not be useful for its intended audience (Laurans et al. 2013). The research presented here responded to three key decision-support motivations. First, the Swedish Environmental Protection Agency (SEPA) released a call for research with the aim to support efforts to meet the expressed national objective of including the value of ecosystem services in decision-making (Ds 2012: 23 2012). Research funded through this call was intended to focus on practical applications of the concept. Second, Kristianstad Biosphere Office was experiencing challenges with brownification and believed that an ecosystem service approach could support a coordination effort to address this complex challenge, which is caused by a combination of processes (Kritzberg et al. 2020), many of which are happening upstream, outside of the reserve boundaries. This motivated Kristianstad Biosphere Office to contact members of the author team to jointly apply for the SEPA funding. Third, members of the author team were keen to test pragmatic, participatory methods for increasing complexity thinking (Preiser et al. 2018) in a Swedish decision-making context, as an understanding for complex adaptive systems is believed to be key for the resilience and long-term sustainability of a social–ecological system (Biggs et al. 2012). This threefold motivation crystallized into a specific decision-support need and the underlying aim of the project as a whole, namely, to develop and test a method using the ecosystem service concept that could increase the understanding for how to address complex, landscape-level sustainability challenges in strategic planning. This aim guided the choice of assessment method and process design. The current paper is focused on the ecosystem service assessment, while a forthcoming paper will discuss the extent to which later steps in the project addressed learning about complex adaptive systems.

## Materials and methods

### Participatory process to locally embed ecosystem service assessment

The research in this paper was conducted through a participatory process with actors from the Helge å catchment. Considering the previously described decision-support need, an ecosystem service bundles analysis was selected as assessment approach as it is based on a relatively simple statistical clustering method, uses publicly available data and can provide a broad overview of conditions in the catchment as well as highlight interactions between services (Queiroz et al. 2015). The relative simplicity of the method makes it both easy to explain to participants and easy to replicate, which encourages engagement and trust in the process (Hamann et al. 2015; Boeraeve et al. 2018). To ensure that a range of relevant provisioning, regulating and cultural services would be considered, we invited representatives from different sectors, including civil servants from local and national agencies, industry associations and civil society organizations with management responsibility and/or a direct claim or interest in the landscape (Table 1 and Appendix S1). The selection was guided by an initial stakeholder scoping done together with the project partners Kristianstad Biosphere Office, who have extensive local knowledge and well-developed networks with actors in the study area. All invited participants had extensive and diverse knowledge about the landscape, be it through working in the forest industry, with nature protection or with strategies to support tourism enterprises. This focus on important local knowledge-holders served our purpose to co-produce an understanding for the current state of and various landscape values in the Helge å catchment. Between 2015 and 2018 we held five workshops, out of which the first three provided the results for this paper. Half of the participants took part in at least two of the first three workshops. The reason for some dropping out was mainly change of job or retirement.

**Table 1:** List of participant affiliations. For further description of the aims and responsibilities of the different organizations, see Appendix S1

Category	Organizations	Number of participants
Municipalities	Kristianstad (including Kristianstad Biosphere Office), Osby, Östra Göinge and Älmhult	6
County boards and national agencies	Skåne and Kronoberg counties and the Swedish Forest Agency	5
Business sector representatives	The Federation of Swedish Farmers, Södra Skogsägarna (the largest forest-owner association in Sweden), Sveaskog (forestry) and Destination Småland (tourism)	5
Civil society organizations	The Swedish Society for Nature Conservation, Sportfiskarna Kronoberg (recreational fishing association) and Jägareförbundet (national hunting association)	4

Before the first workshop, we made a preliminary selection of ecosystem services together with representatives from Kristianstad Biosphere Office. This selection was guided by the ecosystem services framework from the Millennium Ecosystem Assessment (MA 2005) and the list of ecosystem services and their indicators from a previous study (Queiroz et al. 2015), where a similar data analysis was conducted on a comparable Swedish catchment area. The preliminary selection was adjusted to correspond with what our project partners at Kristianstad Biosphere Office deemed most relevant to include, considering the study area, while at the same time maintaining a balance between the categories provisioning, regulating and cultural ecosystem services.

At the first workshop we presented a draft analysis of the spatial distribution of ecosystem services in the catchment. Using these maps as a basis, participants were asked to revise the analysis in facilitated group discussions. They were asked if the distribution looked accurate, according to their knowledge of the study area, and if inaccuracies might be due to the selected indicators being poor proxies of a service. We also discussed if the list of services as a whole was able to represent key characteristics of the landscape, according to what the participants valued and considered important for understanding conditions and processes in the catchment. Discussions were facilitated so that participants representing different types of knowledge and interests could voice their opinion and constructively discuss the selection with each other. Suggested adjustments were summarized for the participants in the end of the workshop, to allow the group as a whole to approve the next steps of the process.

Following this, we performed a new analysis of the selected services by generating a set of ecosystem service bundles. At the second workshop we presented the updated analysis, which resulted in smaller adjustments suggested by the participants in facilitated group discussions. At the third workshop, we presented a final analysis and discussions focused in particular on the emergent distribution of ecosystem service bundles across the Helge å landscape. Throughout the process, workshop participants discussed relevant issues that our analysis did not capture, which served as an important source of information for us when evaluating the approach. For a more detailed description of the workshop design, see Appendix S2.

### Interviews with workshop participants

In order to capture individual reflections on the process and the ecosystem service bundles analysis, we conducted two rounds of semi-structured interviews with the participants, one after the second workshop and one in the end of the 3-year project. The interviews lasted 1 h on average (see Appendices S3 and S4 for interview guides). The interviews were transcribed and coded for emergent themes. We used the data collected through interviews to triangulate results of the ecosystem service assessment (first interview), and also to evaluate the usefulness of our method and its outputs for the participants (first and second interview).

### Mapping and methods for assessing interactions among services

The final ecosystem service assessment included 15 services (see Table 2 in “Results” section) across 42 municipalities in southern Sweden (to ensure the stability of the statistical cluster analysis, the assessment included all municipalities that overlap with the Helge å catchment, as well as all municipalities in Kronoberg and Skåne counties). Based on the methodology used by Queiroz et al. (2015), we selected appropriate indicators for each ecosystem service and retrieved relevant datasets from public databases. These databases include the Swedish Board of Agriculture National Statistics Database, which contains data on agricultural production, the Swedish Land Survey database, which provides various spatial data, as well as national datasets on nature protection and environmental quality provided by the Swedish Environmental Protection Agency. To keep the analysis consistent, we selected indicators reflecting the current capacity of the landscape to generate each individual service, following the conceptual framework by Villamagna et al. (2013). However, indicators representing the landscape capacity were not available for all services that the participants found important to include in the analysis, which is why in some cases we had to select indicators that reflect the flow of a service. The indicators for standing and running water quality and hunting are examples of such compromises. All values for each indicator were normalized by the maximum value in the study area for that particular service, to facilitate comparisons. From this we produced maps of how the individual ecosystem services were distributed. Additionally, we produced maps of the residuals from the regional average, to identify “hot-spot” (higher production) and “cold-spot” (lower production) municipalities for each category of services (Queiroz et al. 2015).

A common comment during the workshops was that representing the individual services like we had done, through relative values within the study area, could in some instances be misleading. By this, the participants did not mean that the data as such was flawed, but that the visualization did not communicate the degree to which the level of ecosystem service generation was satisfactory. In particular, this concern was raised when discussing the indicators for water quality. The even distribution of these regulating services made it “look” like water quality was good, even though poor water quality in the form of brownification was perceived as one of the main sustainability challenges in the study area. To address this shortcoming, we developed goal-oriented societal aspiration indicators for one provisioning, one regulating and one cultural ecosystem service. These indicators show how much a particular service would be requested by people living in a particular spatial unit and moment in time based on a sustainability-oriented societal goal. We then generated maps comparing the current ecosystem services generated with the societal aspiration by using a simple ratio. This step was only done for the 13 municipalities that fall within the Helge å catchment. These maps reflect what ratio of the local societal aspiration for a particular service that could be provided for within a municipality. Comparing aspirations with the current levels of service generation like this can give an indication of the sustainability of current practices and thereby help address sustainability challenges in decision-making. Such normalizations of complex ecosystem service information have been suggested as one way to reduce the cognitive load for decision-makers (Wright et al. 2017). In our case, this analysis helped contextualize the results and facilitated the interpretation of management implications.

To analyze interactions and delineate bundles, we conducted a correlation analysis to identify the existence of potential synergies and trade-offs between ecosystem services (Queiroz et al. 2015). Furthermore, we assessed the interactions among multiple ecosystem services using a cluster analysis. We used the NbClust R package to determine the optimal number of clusters for the current dataset. K-means clustering was used to identify distinct types of bundles, and these were represented by rose-wind diagrams. These diagrams were dimensionless, as all bundles were calculated from the normalized values obtained for each service. All statistical analyses were conducted in R (R Core Team 2013).

## Results

### Participatory iterative selection of ecosystem services and indicators

**Fig. 2:**
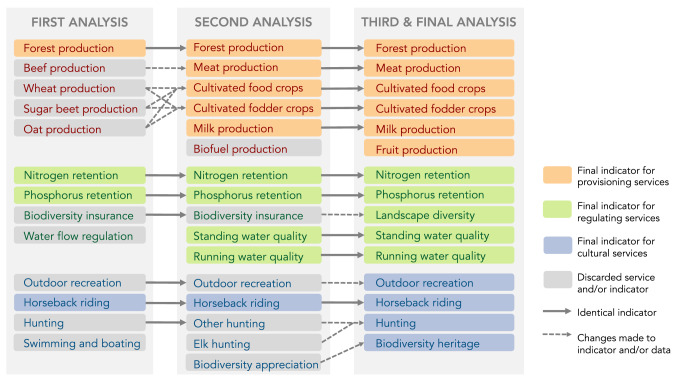
Changes in selection of ecosystem services throughout the three iterations of the participatory process. Services with only colored boxes and solid arrows between iterations stayed identical throughout the whole process (e.g., forest production; nitrogen retention). Services with a gray box and no arrow to the next iteration were dropped from the analysis (e.g., biofuel production; swimming and boating). Services with gray boxes and a dashed arrow to the next iteration have been reformulated, either by changing the indicator or by adjusting the parameters of the input data (e.g., outdoor recreation). In some cases, data from several indicators were merged to represent an aggregate service, to make the selection of services more informative of key characteristics of the landscape and/or to achieve a balance between service categories (e.g., wheat, sugar beet and oat production in the first iteration were split up and combined with additional data to produce cultivated food crops and cultivated fodder crops for the second iteration). In some cases, changes to an indicator motivated a re-naming of an ecosystem service (e.g., biodiversity insurance to landscape diversity). We chose to represent these ecosystem services in the figure as updated versions of the same service (rather than new services) because they were discussed in the workshop and included in the analysis to represent the same general values or important characteristics of the landscape (as perceived by the participants)

For the first workshop we analyzed 13 ecosystem services, in the second 16, and in the final 15. Figure 2 shows how the selection of ecosystem services and suitable indicators to represent them were reformulated between iterations. For example, biofuel production was included in the second iteration, but replaced by fruit cultivation in the last iteration, after discussions about which features really characterize the landscape around Helge å. Another example is the indicator for landscape diversity, which was radically changed after the first round (then called biodiversity insurance), since the first version produced a picture of the landscape that the participants did not recognize. Participants who knew the landscape well could also precisely explain what landscape features (in this case a large lake) that had skewed the results, suggesting that the original input data were not a good proxy for the intended service. However, it was important for the participants to include a service indicating some aspect of biodiversity. Unfortunately, none of the other proxies we explored were salient enough. We therefore landed on a compromise, mapping the diversity of valuable habitats (protected as Natura 2000 sites), with the assumption that a diverse landscape is also likely to support biodiversity. Landscape diversity (or habitat for native diversity) is sometimes (but not always) considered an ecosystem service, so we have grouped it with the regulating services for simplicity. Table 2 lists the services and indicators from the final iteration.

**Table 2:** List of ecosystem services, their indicators and data sources from the final iteration of the ecosystem service analysis. All data for 2014, if not otherwise specified

Ecosystem service	Indicator	Unit	Data source
*Provisioning services*			
Cultivated food crops	Harvested area for crops that are used for food production in relation to municipality area (wheat, rye, oats, barley, rape seed, peas, beans, potatoes, sugar beets)	km^2^/km^2^	Swedish Board of Agriculture National Statistical Database
Cultivated fodder crops	Harvested area for crops that are used for fodder in relation to municipality area (hay and pasture, cereals, maize, potatoes for starch, rape, forage peas, vetch, field beans, sugar beets)	km^2^/km^2^	Swedish Board of Agriculture National Statistical Database
Fruit production	Area of fruit orchards and berries relative to municipality area	km^2^/km^2^	Lantmäteriet (national land survey)
Meat production	Number of meat cows and sheep in relation to municipality area	No./km^2^	Swedish Board of Agriculture National Statistical Database
Milk production	Tons of milk produced in relation to municipality area	Tons/km^2^	Swedish Board of Agriculture National Statistical Database
Forest production	Area of production forest in relation to municipality area	km^2^/km^2^	Lantmäteriet (national land survey) and National Board of Forest
*Regulating services*			
Nitrogen retention	Retention from nitrogen pollution of agricultural land and private sewers. Long-term average for the period 1985–2004, in relation to municipality area.	Average fraction of 1 − (net nutrient load/gross nutrient load)	Swedish Environmental Emissions Data (SMED)
Phosphorus retention	Retention of total phosphorus load. Long-term average for the period 1985–2004, in relation to municipality area	Average fraction of 1 − (net nutrient load/gross nutrient load)	Swedish Environmental Emissions Data (SMED)
Standing water quality	Average water quality score/km^2^ of standing water in the municipality (classes 1–5, from low to high grade)	Average score	Water Information System Sweden (VISS) Database
Running water quality	Average water quality score/km^2^ of running water in the municipality (classes 1–5, from low to high grade)	Average score	Water Information System Sweden (VISS) Database
Landscape diversity	Shannon diversity index calculated from Natura 2000 sites: Each unique combination of main Natura 2000 categories (Coasts and salt affected habitats; coastal and inland sand dunes; fresh water habitats; heaths and shrublands; grasslands; mires; cliffs and caves; forests; bird habitats) were coded as a “species” (= type [72]). Shannon diversity index calculated for each municipality based on the combination of types (number of unique types present & size of sites in relation to municipality area). Data downloaded 2016	Index	Lantmäteriet (Swedish national land survey) and Swedish Environmental Protection Agency
*Cultural services*			
Outdoor recreation	km non-motorized roads (walking paths; biking trails; hiking trails; tractor roads) on natural surfaces (forest and open land [NOT agricultural land]) in relation to municipality area. Data downloaded 2016	km/km^2^	Lantmäteriet (Swedish national land survey)
Horseback riding	Number of horses on horse farm in relation to municipality area	No./km^2^	Swedish Board of Agriculture National Statistical Database
Hunting	Number of boar, deer (3 species) and moose (the latter weight adjusted × 5 [5 = average moose weight/average deer weight]) hunted in relation to municipality area in 2013–2014	No./km^2^	Jägareförbundet (national hunting association), Lantmäteriet (Swedish national land survey) and administrative county boards of Skåne, Kronoberg and Jönköping
Biodiversity heritage	Area of national parks and nature reserves in relation to municipality area. Data downloaded 2016	km^2^/km^2^	Lantmäteriet (Swedish national land survey) and Swedish Environmental Protection Agency

### Distribution of ecosystem services

**Fig. 3:**
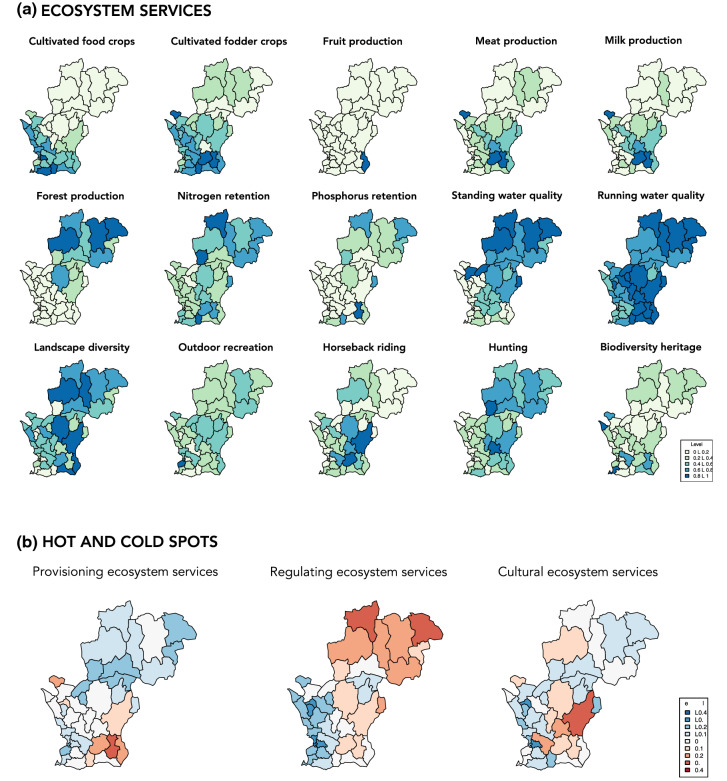
Maps of the 15 ecosystem services as well as hot- and cold-spots for the three service categories: **a** The maps of individual provisioning services show clear spatial patterns, with forest production being high in the north, meat and milk production concentrated in the south-east and cultivated food and fodder crops in the south-west. Food and fodder crops are positively correlated with each other, as are meat and milk production. Forest production shows strong negative correlations with food and fodder crops. Regulating services are generally higher in the north and positively correlated with forest production. Finally, cultural services do not exhibit clear spatial patterns, except for hunting, which is positively correlated with forest production and regulating services, while at the same time being negatively correlated with food production (see Appendix S5 for correlation matrix). **b** The hot- and cold-spot maps show that the south-eastern municipalities stand out as hot-spots for provisioning ecosystem services, the northern municipalities for regulating services and the south-eastern municipalities for cultural ecosystem services. The northern municipalities are cold-spots for provisioning services and the western municipalities for regulating services

Following the participatory selection of the 15 ecosystem services, we mapped their spatial distribution (Fig. 3a). The distribution of provisioning services shows clear patterns, with a concentration of meat and milk production in the south-eastern part of the study area, high levels of cultivated food and fodder crops in the south-west, and forest production mainly in the north. At an aggregate level, the south-eastern municipalities stand out as hot-spots for provisioning ecosystem services (Fig. 3b), meaning that the level is above average in these municipalities.

Similarly, the regulating services nitrogen and phosphorus retention and standing water quality show a clear spatial pattern. They are relatively high in the north and positively correlated with forest production. Running water quality is relatively even across the study area, although slightly higher in the north and east. Landscape diversity is generally low in the west, while the highest values are found in the east (Fig. 3a). In general, the northern municipalities are hot-spots for regulating services, while the western municipalities are cold-spots (Fig. 3b). Cultural ecosystem services do not generally exhibit clear spatial patterns. However, on an aggregate level, the south-eastern municipalities are hot-spots for cultural services.

### Comparison between current and aspiration levels of ecosystem service generation

We developed goal-oriented indicators that represent suggestions for what a sustainable societal aspiration could be. In this case, we selected official health recommendations for meat consumption (Nordic Council of Ministers 2014), the national environmental goal for water quality and an aspiration of meeting a recreational goal for hunting as a cultural activity (that is, at least one animal killed per issued hunting license). The indicators for current generation of meat and water quality have been adjusted from the bundles analysis in order to be comparable with the aspiration indicators. The indicators are listed in Table 3.

**Table 3:** Indicators for societal aspiration and current generation of one ecosystem service in each service category

Ecosystem service	Societal aspiration	Current generation
Meat production	Recommended maximum consumption of red meat multiplied by population density in each municipality to represent the area-average sustainable demand of meat. Recommended maximum consumption of red meat for adults according to health recommendations of the Swedish National Food Agency, adjusted to represent only beef and lamb consumption (i.e., exclude the ratio of red meat consumption made up of pork according to Swedish consumption statistics)	The total amount of slaughtered animals per municipality in kilograms per square kilometer, based on number of animals and average slaughter weight of cows and sheep, respectively. Changed from heads of cattle and sheep (as used for bundles analysis) in order to be comparable with aspiration indicator
	Unit: kg/km^2^	Unit: kg/km^2^
	Data source: Nordic Council of Ministers (2014) and Statistics Sweden Statistical Database	Data source: Swedish Board of Agriculture National Statistical Database and Lantmäteriet (Swedish national land survey)
Running water quality	Total length of river per square kilometer in each municipality. Meant to represent a scaled down version of the national environmental goals for water quality, that is, length of river that should have good or very good quality according to the environmental goals	The length of river assessed as having good or very good water quality per square kilometer. Changed from average quality (as used in bundles analysis) in order to be comparable with aspiration indicator
	Unit: km/km^2^	Unit: km/km^2^
	Data source: Lantmäteriet (Swedish national land survey)	Data source: Water Information System Sweden (VISS) Database
Hunting	Number of hunting licenses sold per square kilometer	Number of animal equivalents killed per square kilometer. This is the same hunting indicator as for the bundles analysis
	Unit: No./km^2^	Unit: No./km^2^
	Data source: Jägareförbundet (national hunting association) and Lantmäteriet (Swedish national land survey)	Data source: Jägareförbundet (national hunting association), Lantmäteriet (Swedish national land survey) and administrative county boards of Skåne, Kronoberg and Jönköping

**Fig. 4:**
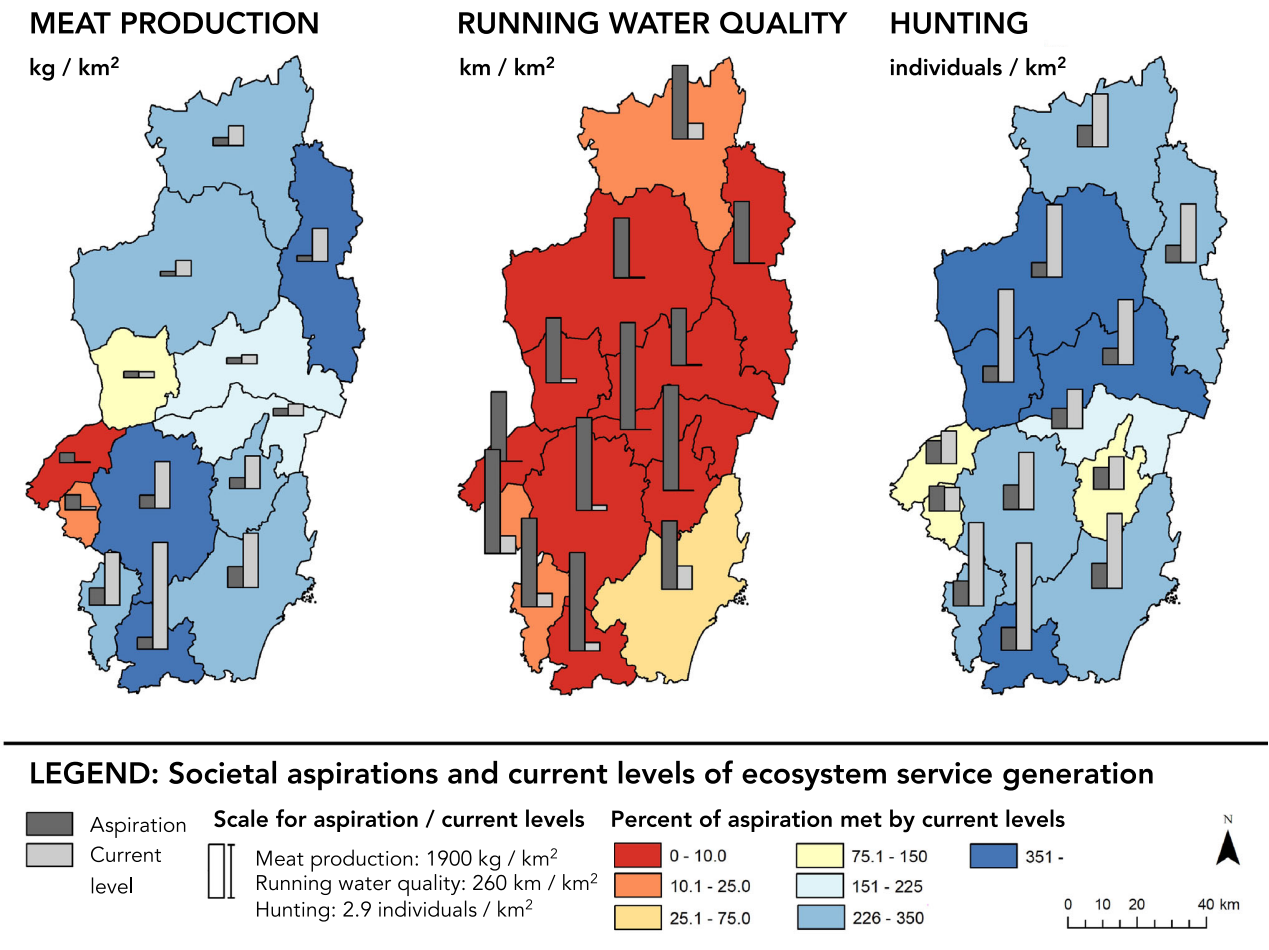
Maps comparing current levels and societal aspirations for one ecosystem service in each category across the municipalities in the Helge å catchment. The bar charts represent the societal aspiration and current level of the three services for each municipality (units specified under the title of each service). The color of each municipality shows societal aspiration in relation to current level, that is, the percentage of the aspiration that has the potential to be met by the current level of the service being generated within each municipality. Values below 100 (red-orange) suggest that the societal aspiration is not being satisfied, while values above 100 (blue) suggest that the current level is higher than the societal aspiration

The maps in Fig. 4 show the comparison between current and aspiration levels of ecosystem services (bar plots) as well as the percentage of the aspiration that could be met by the current levels of service generation (color gradient) in each municipality in the Helge å catchment. Current levels are higher than the aspiration in most municipalities for both meat and hunting. It should be noted that ecosystem service flows in reality tend to be spatially dispersed, meaning that a comparison like this should not be seen as empirical evidence of ecosystem service under- or over-capacity or over-use (Villamagna et al. 2013). Nevertheless, we report the resulting maps as they serve as valuable theoretical entry points and, as such, represent a tool in discussions of local sustainability planning.

### Ecosystem service bundles

**Fig. 5:**
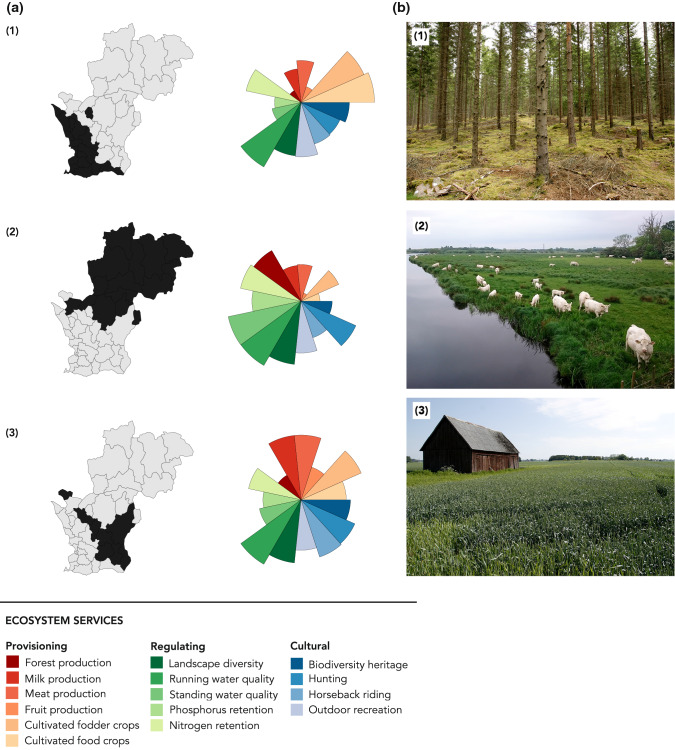
**a** Three clusters of municipalities with their type bundle of ecosystem services. Cluster (1) is forestry-dominated, with high production of regulating services. Cluster (2) has a mixed landscape with high milk and meat production and comparatively high levels of cultural services. Cluster (3) has high production of cultivated food and fodder crops, but generally low levels of all other provisioning and regulating services, and average levels of cultural services. **b** The photographs show characteristic landscapes from the respective clusters (taken by Katja Malmborg)

The cluster analysis identified three distinct clusters of municipalities, based on their typical ecosystem service profiles (Fig. 5). The first cluster, consisting of 15 municipalities in the northern parts of the extended study area, is characterized by relatively high levels of forest production, while all other provisioning services are low. In this bundle the level of regulating services is also high, while the cultural services vary. This is an expected result as the landscape in the north is dominated by forests and the area is sparsely populated.

The second cluster (Fig. 5), consisting of 8 municipalities in the eastern and central parts of the extended study area, has high production of milk and meat, while other provisioning and all regulating services have an average level, and that of cultural services is relatively high. This is also an expected result as the landscape here is more mixed, with a combination of grazing animals and forestry. It also corresponds well with several areas of high biodiversity and recreational values (e.g., Kristianstad Vattenrike Biosphere Reserve), as well as the towns Kristianstad and Hässleholm.

The third cluster (Fig. 5), consisting of 19 municipalities in the western and southern parts of the extended study area, is the least varied in terms of the generation of ecosystem services. The ecosystem service bundle typical of these municipalities is characterized by high levels of food and fodder crops, while the other provisioning services and all regulating services are relatively low. The levels of cultural services are average. This is also expected, as the area is well known for its intensive production of cereal crops and is densely populated, including the city of Malmö. This cluster is less relevant when characterizing the landscape in the Helge å catchment, since only one municipality, Perstorp, overlaps with a very small part of the catchment.

### Workshop participants’ reflections on approach and outputs

The interviews revealed that all participants appreciated coming together in the workshop setting to discuss and share their experiences of, and views on, ecosystem services. Most participants also saw the workshops as a learning opportunity about the ecosystem service concept, which they nowadays frequently encountered in their professional lives. They appreciated how the process increased their understanding for issues relating to, for example, data availability. A participant explained: “We might have thought/…/that [these indicators] aren’t relevant. And from thinking that, to coming up with new ways to represent [the services] is a big step because you had to use data and come up with clever formulations./…/It has been interesting to follow that complexity/…/[and that it] still aims better now than it did with the first attempts./…/[And] from that [the ecosystem service bundles] maybe felt a bit daunting to look at/…/they now feel completely familiar”.

In interviews, all workshop participants confirmed that they recognized the general ecosystem service patterns that emerged from the analysis, with the forested north, the urbanized and agricultural south-west and the multifunctional landscape of the east. A participant with extensive knowledge of the local landscape and biodiversity said: “What you can do, as we’ve done with [the ecosystem service bundles] is to get a general regional landscape overview./…/[About] the picture that it shows, a practitioner would probably not find anything new, one could say that of course that’s how it is—which often is easy to say but to get numbers on it and be able to confirm with data that it actually is the way you think, that is often the tricky part”. While another participant, whose expertise was in business development and regional strategy, explained: “The interesting thing when looking at [the ecosystem service bundles] is really that you can see the difference when you move across the municipalities/…/And I think that is good to have with you. Because people are probably quite happy otherwise, to generalize/…/‘This is what it’s like in southern Sweden, central Sweden’, and then many decisions are based on that. When actually, when looking at it like this [with the ecosystem service bundles], then you can see that a decision would really fall out differently depending on just this small [study area]”.

Several of the participants, especially those working in municipalities and county boards, saw the bundles approach as an effective way to communicate the importance of multifunctional landscapes to other decision-makers, like municipality politicians in the process of setting priorities and assigning budgets. Representatives from two municipalities reported that they are using the ecosystem service bundles to inform the development of strategic sustainability documents (one green plan and one rural strategy). Additionally, as one participant explained: “There is a value for us to be able to say that there are many [ecosystem services] here, and the values those create in terms of resilience and so on. I have talked about that in many contexts”.

Furthermore, some participants mentioned that they appreciated how this approach to ecosystem services also promotes thinking about cross-sectoral cooperation. Interestingly, participants representing the agricultural and forestry sectors appreciated how the bundles approach included provisioning services as part of the analysis. In these participants’ experience, actors in the agricultural or forestry sectors are often left out of discussions about environmental management, or simply framed as the “bad guys” driving the loss of important assets and values in the landscape. As one of them put it: “There was a new perspective in the workshop/…/Because most of the time, if you’re a land owner, farmer or forester, you get something dumped in your lap, for example, ‘how are we now to protect this red-listed species’. While/…/if you turn it around, instead of making land use a problem, [to see it as] an opportunity. That’s how I felt your questions were framed. And it isn’t common that we get it from that perspective nowadays”. They were of the opinion that the bundles approach invited the user perspective in a way that is very useful, but still relatively uncommon, opening up for a broader discussion about potential trade-offs and synergies between production methods, ecosystem health and actors’ motivations behind their landscape management.

## Discussion

### The value of the participatory approach for advancing the bundles analysis

Our spatial assessment of multiple ecosystem services confirms a multifunctional landscape in the Helge å catchment, with some clear geographical differences reflected by three distinct ecosystem service bundles. To a large extent, the agriculture and forestry sectors seem to explain the distribution of provisioning and regulating services, whereas the cultural services are more spread out. The analysis suggests a trade-off between regulating services and agricultural provisioning services, although this trade-off does not seem as strong in Helge å as in Montreal (Raudsepp-Hearne et al. 2010) and Denmark (Turner et al. 2014) where studies using similar bundling approaches have been conducted. And as in the Norrström basin, central Sweden (Queiroz et al. 2015), this trade-off does also not seem to apply for forest and livestock-related provisioning services. Another similarity with the Norrström basin is the existence of municipalities with a relatively diverse ecosystem service bundle, suggesting multifunctional, or mosaic, landscapes (Queiroz et al. 2015). In Helge å, this is represented by the second cluster, located in the east-central part of the study area. However, compared to the two mosaic clusters in Norrström, the east-central municipalities in Helge å have higher levels of cultural services. That is potentially due to a higher population density in this cluster having motivated more infrastructure and protected areas, leading to increased access to nature.

When reflecting over the causes behind the pattern, the participants listed geology and geomorphology as the main biophysical condition, with fertile soils in the south-west creating good conditions for industrialized agriculture. Poorer soils and varied topography in the north has made modernization difficult there, causing a shift from the mixed, small-scale agriculture of the past to a forestry-dominated landscape (Tuvendal and Elmqvist 2011). Market development has exacerbated these conditions. To summarize, our analysis shows patterns that are partly similar to both the trade-offs seen in Denmark (Turner et al. 2014) and the multifunctionality discovered in Norrström (Queiroz et al. 2015), suggesting a landscape in the transition zone between the intense agriculture in continental Europe and the forests of the north.

The participatory approach provided critical improvements in the saliency and credibility of our assessment. As shown in Fig. 2, most of the ecosystem services that were included in the first iteration were either changed or the indicators for them updated to better represent the participants’ understanding for what is important for characterizing the Helge å catchment. Several of the participants knew the used datasets well, which meant that they could not only point out where a service was being misrepresented, but also suggest how the indicator could be improved. We believe that this active engagement in discussions, ranging from selection to data use, was an important contributing factor to how well our analysis later was picked up and used by the participants (discussed further in the next section). This is supported by the interviews, where the quote “[it] still aims better now than it did with the first attempts” serves as an example of the increased credibility and saliency, both of which are important for making knowledge usable (Clark et al. 2016; Boeraeve et al. 2018).

In addition, our approach clearly identified the limitation of only looking at current levels of ecosystem service generation. Particularly the relatively even water quality across the study area was problematic for the participants, since the visualizations made it seem like water quality was sufficient and this did not correspond to the participants’ perception of brownification being one of their main sustainability challenges. Following an important discussion around this, we decided to add the societal aspirations analysis. While the aspirations analysis could not be carried out across the full range of services, we see a substantial potential here for method development to really operationalize the ecosystem service concept. Similar ideas have been surfaced before in demand (Burkhard et al. 2012; Baró et al. 2016) and preference analyses (Martín-López et al. 2012). Including societal aspirations (or demand/preference) for ecosystem service generation in analyses extends the reach of relevant sectors beyond environmental protection, land use planning and agriculture, to also include public health, business and education in a meaningful way. This added relevance was also brought up in some of the interviews, highlighting the assessment’s potential to inform more integrated management strategies.

In summary, we find that the participatory approach was critical in making the ecosystem service bundles analysis relevant to actors’ needs and perspectives. In the end, this process gave us a balanced selection of relevant services and comparable indicators, analyzed though an accurate and reliable enough method to meet the intended decision-support need (Barton et al. 2018). In this case, that meant awareness-raising by increasing the understanding for landscape-level sustainability challenges and the value of multifunctionality (Gómez-Baggethun and Barton 2013) and through this, conceptual and strategic knowledge used by the participants when communicating and negotiating with other actors in their organizations (McKenzie et al. 2014). The resolution of this analysis is too coarse for some decision-making purposes, such as spatial planning. The somewhat mixed character of the indicators also makes them unsuitable to use in, for instance, monetary valuation. Yet, the chosen resolution allowed us to include a wider range of indicators, particularly those of a socially defined nature, which would not be available at a finer resolution. By assessing ecosystem services based on more diverse data than just land cover, we can create a nuanced, social–ecological understanding of the landscape suitable for highlighting existing values, areas in need of more attention and opportunities for well-informed prioritizations to be made about future, more detailed and costly sustainability analyses.

The participatory process, however, was far from trivial. It required cooperation between actors with knowledge of, expectations on, and attachment to the landscape, and experts with knowledge in ecosystem service analysis. Even in a data-rich context like Sweden, it proved to be a challenge to find publicly available, relevant and comparable indicators, at the right resolution, for the 15 different services. While people largely recognized themselves in the emerging picture of the landscape, there were also some important gaps in the analysis that were directly related to data availability, particularly for cultural ecosystem services. An example was biodiversity appreciation, which could not be represented in a satisfactory way according to the participants, and was dropped from the analysis. Those data gaps will be important to cover to operationalize the full spectrum of the ecosystem service concept for strategic sustainability planning and related decision-support contexts. National and municipal datasets of cultural and social expressions of human–nature interactions could be added to already existing socioeconomic survey programs that are run by Statistics Sweden or other agencies. Public participation geographical information systems (PPGIS) also show promise (Samuelsson et al. 2018), but these would have to be conducted consistently over larger regions for the data to be compatible with the resolution and scale of our bundling approach.

Even with these challenges, however, we think the effort was worthwhile. Since one important motivation for testing this method was to help provide means for coordinating action to address a complex landscape-level sustainability challenge, the outputs of the analysis have to reflect the diverse values and priorities that local actors see as important in the landscape. Without this type of knowledge co-production process, there is a risk that scientists or managers let data availability fully determine the course of analysis and as a consequence miss important local concerns.

### Participatory assessment of ecosystem service bundles as an asset for strategic sustainability planning

The ecosystem service concept is frequently used in both scientific and popular debates, and decision-makers and managers in Sweden are being encouraged to use it. However, there are many remaining question marks regarding its operationalization (Beery et al. 2016). This type of participatory process, focused on understanding a landscape from an ecosystem service bundles perspective, currently seems to be attractive for many actors involved in landscape management (Brunet et al. 2018). Through our participatory approach, we have been able to show that an ecosystem service bundles analysis similar to that used by Raudsepp-Hearne et al. (2010) and Queiroz et al. (2015) can produce useful knowledge for local practitioners. As exemplified by some of the quotes in the Results, the participants have come to value the analysis as material they can draw on, both as a convincing terminology and as strong visuals for communication, empowering them in their negotiations about landscape management and strategic sustainability planning with other actors in their organizations (McKenzie et al. 2014). This is made most clear by the fact that the ecosystem service bundles already have been used in the process leading up to developing strategic sustainability plans in two municipalities in our study area, as reported by participants in our project. More generally, the participants reported that, as a concept, the ecosystem service bundle was an effective way to characterize a landscape and a tool to communicate the value of multifunctionality. In addition, two other aspects emerged as particularly useful with the concept, relating to the meeting between different actors and to the co-production of knowledge.

First, participants saw ecosystem services as a concept to meet around. In fact, we believe that the ecosystem service bundles have worked as a “bridging concept” (Baggio et al. 2015). The process of producing them was a useful entry point that could gather people who care about the landscape but have different perspectives and priorities. This was appreciated by all workshop participants. Especially telling was that the participants involved in agriculture and forestry, and who have a strong attachment to the landscape, appreciated an arena where they could be listened to, and not just portrayed as the “bad guys”. Partly, this was likely due to the process design. However, the fact that the bundles brought in the provisioning ecosystem services as part of the discussion around sustainability placed these participants in a position of knowledge-holders. This differs from similar discussions where actors such as farmers and foresters are placed in a position of having to defend their “environmentally harmful” activities.

Second, for us as researchers, “the way in” and the reason we could initiate this process was that we could offer a scientifically sound analysis of the ecosystem services in the study area. This led to a willingness among the participants to jointly explore these issues. Simply being educated about the concept, using a conceptual representation of an ecosystem service bundle (which would have required less resources), would not have had the same empowering effect. An important aspect of the ecosystem service bundles was how they captured the local landscape, visualizing empirical data from the area, allowing participants to, for example, “be able to say that there are many [ecosystem services] here, and the values those create in terms of resilience”. This strengthened the usefulness of the bundles as tools in the participants’ strategic knowledge use when promoting more sustainable decision-making in their broader organizations (McKenzie et al. 2014). In its function as a bridging concept, the process of co-producing the ecosystem service bundles has also enabled approaching more non-conventional issues for sustainability work, such as complexity thinking (Preiser et al. 2018). It created a shared understanding of the system between the diverse actors, which has been noted in previous participatory ecosystem service assessments as well (Boeraeve et al. 2018). The fact that the bundles represent a wide range of knowledge on different services has further contributed to the effectiveness of this bridging concept, as everyone has had something to contribute to the discussions. The end product therefore truly is the result of a collaborative learning journey, embracing the complexity of the social–ecological system. We believe that this was an important step for future deliberations, paving the way for further discussions about sustainability.

## Conclusions

Operationalizing the ecosystem service concept for addressing complex sustainability challenges requires a social–ecological lens and legitimate participatory processes for co-management. For supporting initial coordination and setting of priorities, the approach also needs to be pragmatic, without relying on complicated data collection or analysis methods. By conducting an ecosystem service bundles analysis using a participatory approach, we have been able to show that such an assessment can be informative yet feasible. While we have identified some important challenges relating to the representation of cultural values in the landscape, our analysis of ecosystem service bundles using publicly available data proved to be a fruitful way towards operationalization. Our participatory analysis resulted in a more salient and legitimate picture of the multifunctional landscape in the Helge å catchment. The co-produced knowledge has been useful for the participants and the ecosystem service bundles also functioned as a bridging concept, serving to create a shared understanding of the social–ecological system among the participants. The latter is important for enabling coordination between diverse actors and essential for strategic sustainability planning, especially to identify joint future aspirations and designing innovative management that will help us navigate complex sustainability challenges.

## Electronic supplementary material

Below is the link to the electronic supplementary material.

Supplementary material 1 (PDF 191 kb)
