# Correction to: Operationalizing ecosystem service bundles for strategic sustainability planning: A participatory approach

**DOI:** 10.1007/s13280-020-01406-9

**Published:** 2020-11-15

**Authors:** Katja Malmborg, Elin Enfors-Kautsky, Cibele Queiroz, Albert Norström, Lisen Schultz

**Affiliations:** 1 Stockholm Resilience Centre, Stockholm University, Kräftriket 2B, 10691 Stockholm, Sweden; 2 Prosperous Planet, Sveavägen 131, 11346 Stockholm, Sweden

## Correction to: Ambio 10.1007/s13280-020-01378-w

In Fig. 5 in the original publication, cluster 1 was mistakenly labelled as 2, cluster 2 as 3 and cluster 3 as 1. The updated Fig. 5 is provided in this correction.

**Fig. 5:**
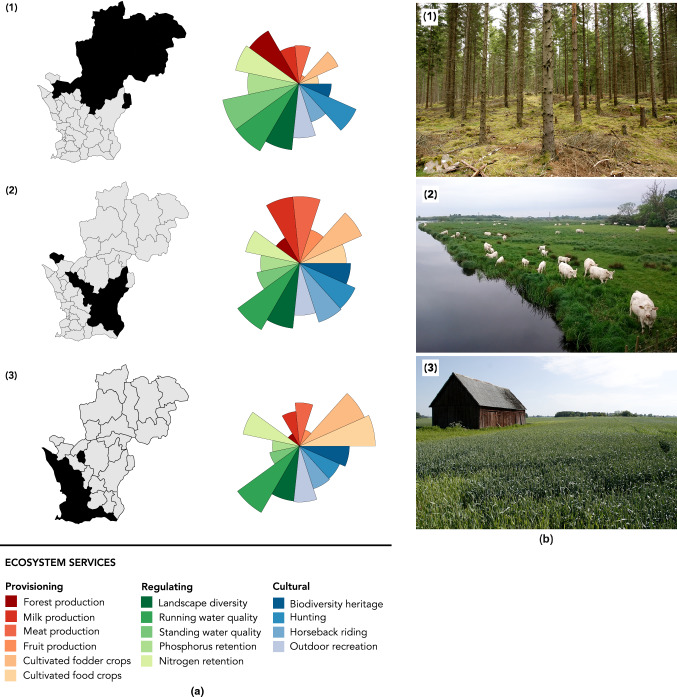
**a** Three clusters of municipalities with their type bundle of ecosystem services. Cluster (1) is forestry-dominated, with high production of regulating services. Cluster (2) has a mixed landscape with high milk and meat production and comparatively high levels of cultural services. Cluster (3) has high production of cultivated food and fodder crops, but generally low levels of all other provisioning and regulating services, and average levels of cultural services. **b** The photographs show characteristic landscapes from the respective clusters (taken by Katja Malmborg)

