# Supplementary material for: Operationalizing ecosystem service bundles for strategic sustainability planning: A participatory approach
Source: Ambio. 2020 Sep 18;50(2):314–31. doi: 10.1007/s13280-020-01378-w (PMC7782664; doi:10.1007/s13280-020-01378-w)

Ambio

Electronic Supplemetary Material
This supplemetary material has not been peer reviewed.

Title: **Operationalizing ecosystem service bundles for strategic sustainability planning: A participatory approach**

Katja Malmborg, Elin Enfors-Kautsky, Cibele Queiroz, Albert Norström, Lisen Schultz

APPENDIX S1 - Aims and responsibilities of organizations that workshop participants are affiliated to

Name/type of organization	Aims and responsibilities
Municipality	Swedish municipalities are responsible for a wide range of societal services and have a high degree of independence with regards to decision-making and planning. Responsibilities of relevance for landscape management and sustainability include physical and strategic planning, construction and (some) infrastructure, environmental protection, water, sanitation and education.
County board	A county board is the public authority responsible for the state management in each of Sweden's 21 counties. Each county contains several municipalities. County boards all have the aim to promote sustainable environmental, social and economic development in their regions. They act as a link between the national and local levels, and one of their main responsibilities is to coordinate between different local, regional and national authorities. They are also responsible for regional planning and for providing expertise and support to municipalities, for example with regards to environmental protection. They also administer several state and EU subsidies for agriculture and environmental protection.
Kristianstad Biosphere Office	Office within Kristianstad municipality coordinating and managing the work in the UNESCO Kristianstad Vattenrike Biosphere Reserve. Responsibilities include environmental management and monitoring as well as education and promotion of outdoor recreation. They function as a bridging organization and coordinator between the different actors that are part of the biosphere reserve, including land owners, farmers and civil society organizations.
Swedish Forest Agency	The national authority in charge of implementing the national forest policy. Their main function is to promote the two main objectives of the policy: economically and ecologically sustainable forestry. They cooperate with representatives from the forest industries and environmental sectors.
The Federation of Swedish Farmers	Association organizing actors within the green sector (agriculture and forestry). Responsibilities include connecting its members with each other, supporting business development for its members, and lobbying for local to national policies that benefit the green sectors and sustainable rural development.
Södra Skogsägarna	Economic association connecting forest owners in southern Sweden. It provides forestry-related services to its members, including planting, processing wood and pulp production.
Sveaskog	State-owned forestry company managing forests owned by the state. Largest forest owner in Sweden.
Destination Småland	Company owned by Kronoberg region, focusing on developing and marketing Kronoberg county as a tourism destination. Cooperates with and coordinates between other actors in the tourism industry in the region.
The Swedish Society for Nature Conservation	The largest environmental protection civil society organization in Sweden. They focus on issues such as climate change, oceans, forests, agriculture and toxins. Active from the local to national level with activities from education to lobbying.
Sportfiskarna Kronoberg	Civil society organization focusing on fishing and care for fish stocks and water body health.
Jägareförbundet	Membership organization for hunters in Sweden. Their focus is on both hunting and wildlife care. They also have official responsibilities with regards to some aspects of hunting regulation and wildlife care, given to them by the Swedish government.

APPENDIX S2 – Description of process and workshop design

The participatory ecosystem service assessment of this paper was part of a longer participatory resilience assessment process consisting of five workshops between 2015 and 2018. The process had a three-fold motivation. First, an identified policy need that led the Swedish Environmental Protection Agency to fund six research projects (including the one that resulted in the current paper) to conduct research on how to operationalize the ecosystem service concept for a Swedish decision-making context. Second, an expressed need from the Kristianstad Vattenrike Biosphere Reserve to find methods and tools to address a complex landscape-level environmental challenge. Finally, an interest from the side of the research team to test participatory methods to stimulate a complexity-based systems perspective among participants and tools for how to include this in strategic sustainability planning.

In response to these three motivations, the process was designed to meet two goals. First, to generate usable knowledge and tangible outputs, such as strong visuals, about the state of ecosystem service provision in the Helge å catchment through a participatory process with a diverse set of participants. The current paper focuses on discussing the outcomes of this goal. The second goal, to stimulate a complexity-based systems perspective among participants and use this in strategic sustainability planning, will be the focus of a forthcoming publication. The process was divided into two phases, loosely corresponding to the two goals. The ecosystem services assessment phase mainly took place during workshops 1-3. The results of these workshops and connected interviews are presented in the current paper. The strategic planning phase mainly took place during workshops 3-5, and will be described further in a forthcoming publication.

Each of the five workshops ran over a full day in the visitors' center of Kristianstad Vattenrike Biosphere Reserve. Each of the workshops were structured around one or two main themes. They started with capacity building components, such as short lectures on underpinning concepts and relevant research. The introduction lecture in the first workshop introduced the ecosystem service concept, some of its history and how we in the research team connected it to sustainability. This included showing conceptual figures that were meant to guide our work together. These figures included 'the doughnut' (Raworth 2017), where sustainability is defined as development within a safe and just operating space where human social and cultural needs are met, while planetary boundaries (Steffen et al. 2015) are not exceeded. For landscape governance, we suggested that sustainability, as understood through the 'doughnut', could be approached through an ecosystem service bundles lens. We also explained our mandate as researchers, that is, the objective of our funding from the Swedish Environmental Protection Agency. In later workshops, this introduction was repeated, in addition to the short lectures about concepts and research of particular relevance for the themes and exercises of the day.

Following the introductions and capacity building components, we had discussions and participatory exercises both in smaller groups and with all workshop participants. After every workshop, the research team processed and updated the exercise outputs based on scientific literature and information from interviews with the participants. The updated outputs, for example new visuals or brief text summaries, were then brought back to the group in the next workshop, making up the starting point or discussion material for the following steps in the process. This meant that the assessment became iterative, for example that all outputs from the process were discussed during at least two consecutive workshops. This approach gave the participants a chance to both repeat what they had learned and to question and change the output.

The focus of the first three workshops (the ecosystem service assessment phase) was as follows: The first workshop introduced the ecosystem service concept to the participants, presented the initial ecosystem service selection and started the discussion about which services to include in the assessment and how to represent them. The second workshop presented the updated ecosystem service selection and their indicators, as well as a first version of the ecosystem service bundles. The third workshop presented the final selection of ecosystem services, their indicators and the resulting ecosystem service bundles. Throughout the process, the research team facilitated discussions about the service selection, the input data and indicators, as well as the ecosystem service

bundles and potential causes behind the distribution of both individual services and the ecosystem service bundles.

References:

Raworth, Kate. 2017. A Doughnut for the Anthropocene: humanity's compass in the 21st century. Lancet Planetary Health 1:e48-e49.

Steffen, Will, Katherine Richardson, Johan Rockström, Sarah E. Cornell, Ingo Fetzer, Elena M. Bennett, Reinette Biggs, Stephen R. Carpenter, et al. 2015. Planetary boundaries: Guiding human development on a changing planet. Science 347:1259855.

APPENDIX S3 – Interview guide, 1st round

Part 1: Experience of previous workshops
- What do you remember from the workshops?
- What was new? (What did you learn?)
- What was frustrating?
- What had an impact? (In your work / practice / way of thinking)

Part 2: Ecosystem services in the Helge å catchment
Interviewees were provided with the individual ecosystem service capacity maps and a list describing the chosen indicators for each service.
- Take a look at the maps and indicators. Do you have any initial reflections or comments?
- Which 5 ecosystem services do you influence the most? How? (In your job? As an individual?
- Which 5 ecosystem services do you use the most? How? (In your job? As an individual?)

Part 3: Ecosystem service bundles in the Helge å catchment
Interviewees were provided with the ecosystem service bundles maps. Interviewer gave very brief description of the characteristics of the bundles with regards to ecosystem service capacity.
- What produces these ecosystem service bundles? (Reflect on all bundles if you feel comfortable with that, or focus on one. You can think about biophysical processes, actors [individuals, organizations, agencies], laws and regulations, etc.)
- Which are the main drivers changing the bundles? Are any of these drivers such that they could make a bundle disappear? (Reflect on all bundles if you feel comfortable with that, or focus on one. You can think about biophysical processes, actors [individuals, organizations, agencies], laws and regulations, etc.)

APPENDIX S4: Interview guide, 2nd round

Part 1: General reflections of workshops
- What do you remember from the workshops you participated in? (Speak freely. Was there anything particularly interesting? Or frustrating?)

Part 2: Exercises and outputs from the workshop process
Interviewees are shown print-outs of photographs from exercises and all assessment outputs. Print-outs: (a) Timeline, (b) Ecosystem services, capacity-aspiration, and bundles, (c) Conceptual system diagrams, (d) Future vision and drivers, (e) 3 key challenges, (f) Strategies (etc.).
- How have these exercise and outputs been useful? (Focus on those that you remember most and/or found most interesting.)

Part 3: The participatory assessment process as a whole
- How did you experience the participatory assessment process as a whole? (Reflect over: Dialogue with other participants, cooperation between participants and research team, the research-focused introductions during each workshop, set-up of exercises, and other.)
- Have you been able to use any of the exercises or what you learned from them in your job? How?
- Do you think you could use anything of it in your job in the future? What? How?
- Is there anything you would have liked to use in your job, but that is impossible to use in the current situation? If so, what and how?

Part 4: General reflections about ecosystem services
- Has this work changed the way you perceive ecosystem services?
- What do you see as the usefulness of the ecosystem service concept?
- Is there anything you would like to add?

APPENDIX S5 – Correlation matrix of individual ecosystem services

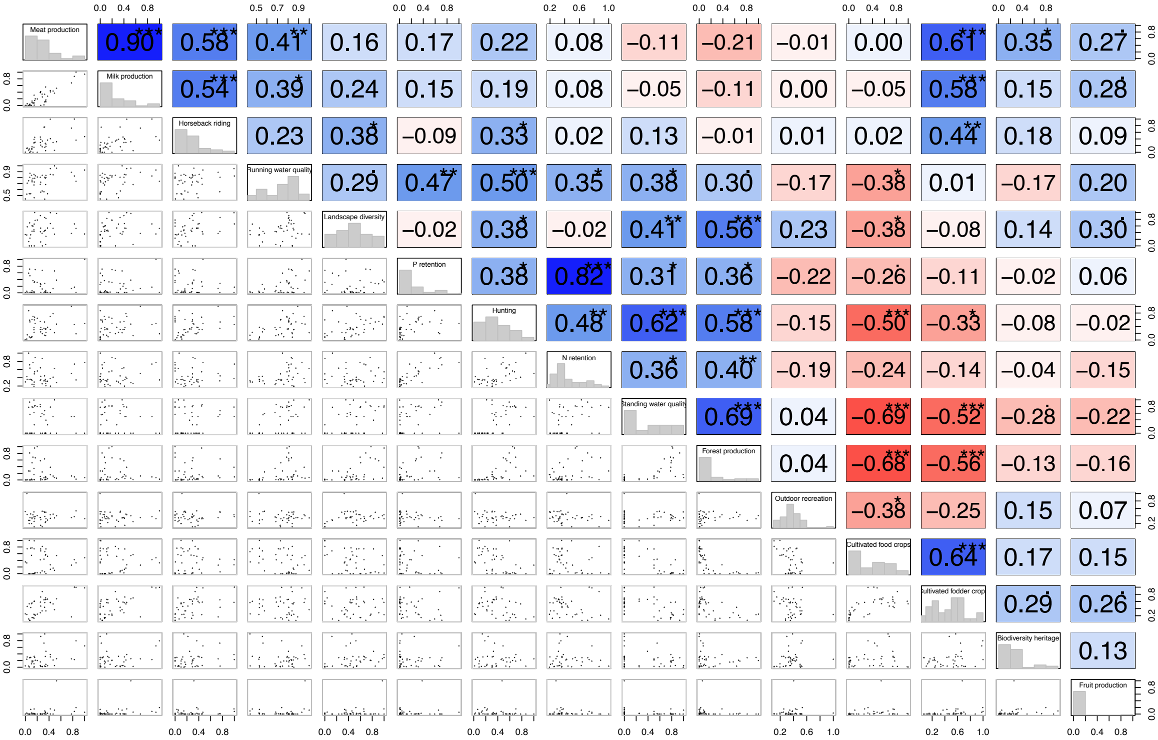

Supplement: Supplementary file 1 — Supplementary material 1 (PDF 191 kb) [file 13280_2020_1378_MOESM1_ESM.pdf]
